# Emerging role of hypothalamus in the metabolic regulation in the offspring of maternal obesity

**DOI:** 10.3389/fnut.2023.1094616

**Published:** 2023-02-01

**Authors:** Jingyi Zhang, Sujuan Li, Xiaoping Luo, Cai Zhang

**Affiliations:** Department of Pediatrics, Tongji Hospital, Tongji Medical College, Huazhong University of Science and Technology, Wuhan, China

**Keywords:** maternal obesity, offspring, hypothalamus, metabolism programming, energy homeostasis

## Abstract

Maternal obesity has a significant impact on the metabolism of offspring both in childhood and adulthood. The metabolic regulation of offspring is influenced by the intrauterine metabolic programming induced by maternal obesity. Nevertheless, the precise mechanisms remain unclear. The hypothalamus is the primary target of metabolic programming and the principal regulatory center of energy metabolism. Accumulating evidence has indicated the crucial role of hypothalamic regulation in the metabolism of offspring exposed to maternal obesity. This article reviews the development of hypothalamus, the role of the hypothalamic regulations in energy homeostasis, possible mechanisms underlying the developmental programming of energy metabolism in offspring, and the potential therapeutic approaches for preventing metabolic diseases later in life. Lastly, we discuss the challenges and future directions of hypothalamic regulation in the metabolism of children born to obese mothers.

## 1. Introduction

It has been established that maternal obesity is a significant predictor of offspring health both in childhood and adulthood. Maternal obesity greatly increases the risk of metabolic diseases such as obesity and type 2 diabetes mellitus in offspring [1–3]. Maternal programming has been proposed to have far-reaching implications on the long-term health of offspring, changes their susceptibility to metabolic disorders, cardiovascular disease, neurodevelopmental diseases and kidney injury [4–7]. The mechanism underlying metabolic disorders in offspring involves intrauterine metabolic programming in regulation of hypothalamic energy homeostasis [4].

Maintaining energy homeostasis requires a balance between energy expenditure and energy intake. Substantial evidence indicates that hypothalamus is critical both in energy expenditure and energy intake by integrating endocrine system and nervous system [8]. Moreover, it is a prime target of developmental programming by maternal nutritional condition [4, 9]. Intrauterine metabolic programming in the offspring hypothalamus has been linked to lifelong diseases, including obesity, metabolic syndrome and neurodevelopmental disorders [5, 10]. Although a growing body of evidence has demonstrated the crucial role of hypothalamic regulation in the metabolism of offspring exposed to maternal obesity, additional investigations are still needed to clarify the underlying mechanisms.

Here, we reviewed the developmental regulation of the hypothalamus, the pathways of hypothalamic regulation in energy homeostasis, the possible mechanisms underlying the developmental programming of energy metabolism in offspring, and the potential therapeutic approaches for preventing metabolic diseases later in life.

## 2. Developmental regulation of hypothalamus

Neuronal development in the hypothalamus consists of two stages: the differentiation and migration of neurons, and the subsequent formation of functional networks (the formation of neuronal projections and synapses) [11]. The hypothalamus arises from cells of the diencephalon. In mice, hypothalamus development begins at the embryonic day 9.5 (E9.5). During E16.5–E18 cells from hypothalamic ventricular zone (HVZ) migrate, differentiate and then form each hypothalamic nucleus, including highly proliferative progenitor cells in arcuate nucleus (ARC) [12]. ARC is crucial in maintaining energy hemostasis. There are two types of neuron populations in ARC to play a leading role in feeding behavior. One expresses the orexigenic neuropeptides agouti-related peptide (AgRP) and neuropeptide Y (NPY), and the other expresses the anorexigenic peptides proopiomelanocortin (POMC), which together form the melanocortin system [12, 13]. *POMC* expression commences at E10.5 in the vast majority of cells in the developing ventral hypothalamus [14]. At E14.5 to E18.5, however, immature neurons gradually lose the expression of *POMC* and differentiate to NPY/AgRP neurons or alternative cell types [15, 16]. The ARC neuronal projections are immature at birth but develop postnatally during lactation, and the projections to the paraventricular nucleus (PVH) take place on postnatal(P) day 8–10 and to the other nuclei on P12–16 [17, 18]. Differently, the critical period of hypothalamic development is complete during fetus life in humans and nonhuman primates [19]. Reports on human fetal suggest that early hypothalamic neurogenesis occurs limitedly to the ninth and tenth week of pregnancy, and the differentiation of periventricular zone structures takes place during mid and late pregnancy, including suprachiasmatic, arcuate, and paraventricular nuclei [20].

A series of factors are involved in the proliferation and differentiation of hypothalamic progenitor cells. Important among them is the Notch-Hes1/5-Mash1-Ngn2/3-Nhlh2/PC1 pathway. Notch receptor anchoring to its ligand activates the Notch signaling pathway [21]. The repressor Hes1 is suppressed and mammalian achaete scute homolog-1 (Mash1) is upregulated when separated from their ligands. Mash1 is essential for POMC differentiation through downstream factors including neurogenin 2/3 (Ngn2/3) and nescient helix–loop–helix 2/prohormone convertase 1 (Nhlh2/PC1) [14]. Nhlh2, which is regulated by Ngn3, mediates the expression of PC1, which induces the proteolytic cleavage of the POMC precursor into melanocyte-stimulating hormones (MSH) [18, 22].

Metabolic hormones, such as leptin, ghrelin and insulin, which reflect alterations in the nutritional environment, can further influence the development of hypothalamus. Rats with reduced central leptin sensitivity have a decreased density of ARC projections in the PVH and abnormal dendrite morphology in the ventromedial hypothalamus (VMH), which appears to be the result of leptin’s inability to directly stimulate neurite outgrowth from ARC neurons [23]. Leptin influences ARC neuronal axon growth by modulating signal transducer and activator of transcription 3 (STAT3) signal [18, 24]. Investigations in mice also revealed the significant negative action of elevated ghrelin on the development of ARC projection, and deeper investigation found the underlying mechanism is associated with STAT3 signal, which implied an interaction with leptin [25]. Meanwhile, insulin promotes neurogenesis on fetal hypothalamic progenitor neurospheres and changes hypothalamic neuronal amounts. Insulin also has a neurotrophic effect and promotes neurite outgrowth to maintain connections hypothalamic nucleus [11, 26]. However, the definitive mechanisms underlying the programming of neuroendocrine hypothalamic networks remain poorly understood. Moreover, adenosine 5′-monophosphate (AMP)-activated protein kinase (AMPK), a cellular sensor of energy availability, also plays an important role in hypothalamic development. Dephosphorylated AMPK regulates the Notch pathway by affecting the transcription of basic helix–loop–helix (bHLH) genes (including genes such as Hes1/Hes5), thereby influencing the differentiation of hypothalamic neurons and altering the NPY/POMC neuron ratio [9]. In addition, brain-derived neurotrophic factor (BDNF) is a critical gene in the regulation of synaptic plasticity, neural circuit development, and energy metabolism regulation [27, 28].

All of the aforementioned factors determine the function of hypothalamus ARC during embryonic development, ultimately influencing the lifetime metabolism health.

## 3. Hypothalamic regulations of energy homeostasis

The metabolic regulation of hypothalamus is accomplished primarily by hypothalamic nuclei perceiving and integrating metabolic signals from the periphery [29]. Each nucleus contains a highly diverse population of interconnected neurons and glial cells that are interconnected. Key to this regulatory function is the melanocortin system, which locates in ARC and projects to PVH and other brain regions to regulate feeding behavior and energy expenditure further. [18, 30] Activated POMC neurons result in decreased energy intake by regulating appetite and feeding habits, while AgRP neurons are activated to induce feeding, inhibit energy expenditure, and regulate glucose metabolism [8].

POMC neurons and AgRP neurons regulate energy homeostasis by neurotransmitters secreted from their synaptic terminals. POMC neurons are activated under conditions of high energy availability. After receiving positive energy signals such as elevated circulating levels of insulin and leptin, POMC neurons initiate the production of POMC peptides. POMC peptide is processed to form various peptides, including ɑ-MSH. MSH is released from the synaptic terminals of POMC neurons and binds to melanocortin receptors MC4R on neurons in the PVH to inhibit food intake further. On the contrary, AgRP/NPY neurons are activated by increased energy consumption [8, 18]. AgRP/NPY neurons inhibit POMC neurons directly by releasing gamma-aminobutyric acid (GABA), antagonize central MC4R signal and counteract the anorectic effect of α-MSH *via* the release of AgRP, thereby positively regulating feeding behavior [31, 32]. PVH neurons are part of an energy homeostatic circuit that projects to autonomic centers in the hindbrain, such as the solitary tract nucleus (NTS). In order to maintain energy homeostasis, the NTS process appropriate responses to satiety signals and engage in adaptive eating behavior [8, 18].

**Figure 1:**
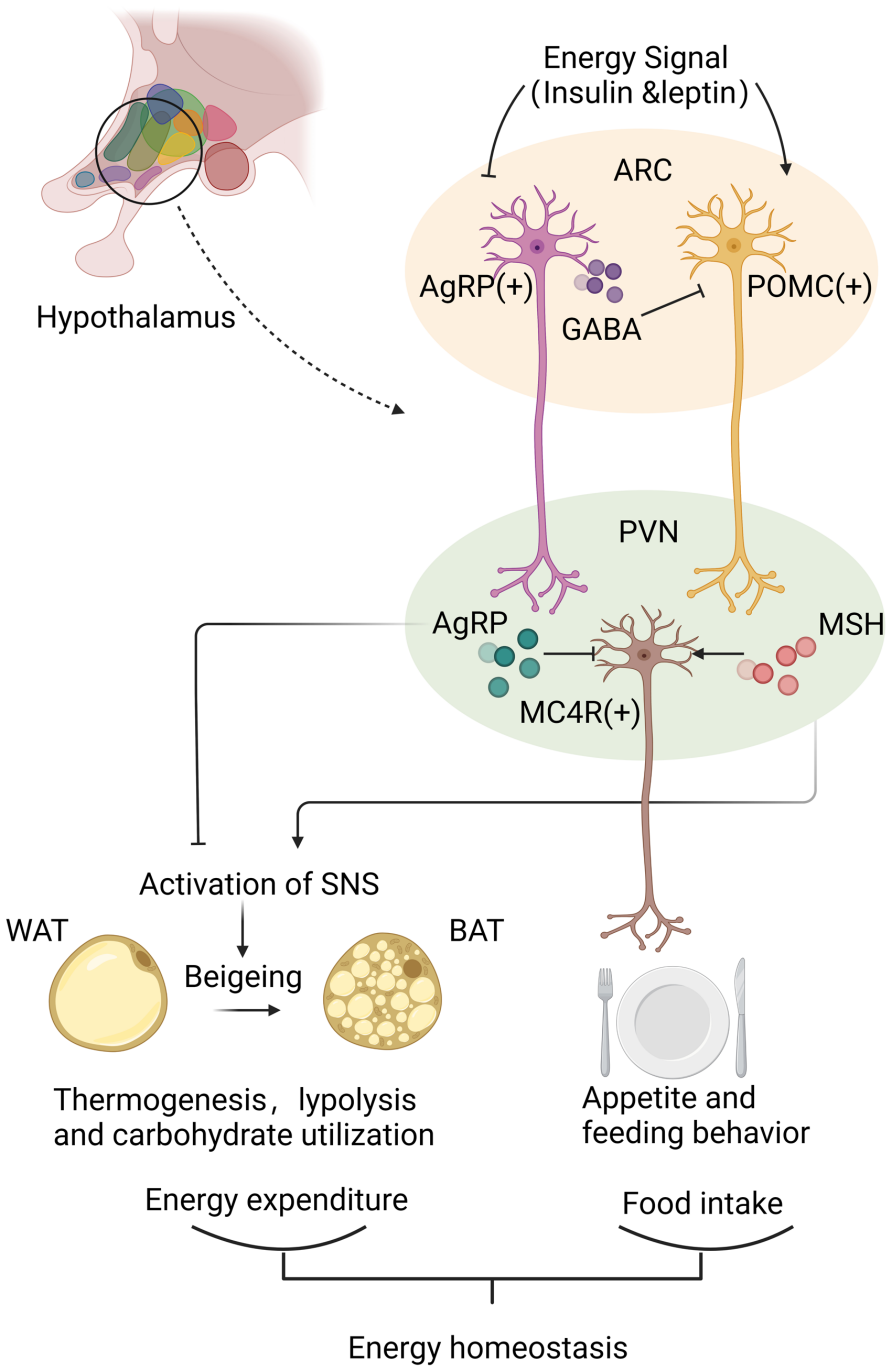
Hypothalamus achieves the energy homeostasis mainly *via* neurotransmitters secreted from synaptic terminals of agouti-related peptide (AgRP)/neuropeptide Y (NPY) neurons and proopiomelanocortin (POMC) neurons in arcuate nucleus (ARC). Under conditions of high energy availability, POMC neurons are activated to produce MSH, which binds to MC4R in the paraventricular nucleus (PVH) to inhibit food intake and increase peripheral energy expenditure through promoting beigeing of white adipose tissue (WAT) and the thermogenesis of brown adipose tissue (BAT). Instead, under conditions of low energy availability, AgRP/NPY neurons release GABA to inhibit POMC neurons directly, and produce AgRP to antagonize central MC4R signal, thereby positively regulating feeding behavior and decreasing the thermogenesis of WAT. By analyzing energy availability and regulating energy intake and expenditure, hypothalamus maintains of energy homeostasis.

Besides regulating appetite, ARC neurons also affect peripheral energy expenditure. POMC neurons facilitate the beigeing of white adipose tissue WAT; beige the conversion of WAT to brown adipose tissue (BAT) and the thermogenesis of BAT [33]. AgRP activation elevates peripheral carbohydrate utilization and reduces lipolysis and suppresses the thermogenic program of WAT [30]. By regulating the activation of the sympathetic nervous system, ARC neurons are known to increase thermogenesis in adipose tissue [30, 33]. Further studies suggested the mechanism could be related with protein tyrosine phosphatase 1B (PTP1B) and T cell protein tyrosine phosphatase (TCPTP) or the sirtuin family, all of which participate in the leptin signal transduction pathway in hypothalamus [33–35] (Figure 1).

## 4. The impact of maternal obesity on the hypothalamus of the offspring

A study in children aged 7–11 years revealed that maternal Body Mass Index (BMI) during pregnancy was positively associated with the hypothalamic response to glucose in children, which predicted the increases in BMI of the children after 1 year [36]. It highlighted the important role of hypothalamic alteration in the energy metabolism of offspring exposed to maternal obesity prior to the onset of obesity. Animal studies provided additional evidence. Offspring born to high-fat diet-fed mothers exhibit induced expression of NPY and reduced expression of POMC in hypothalamus, resulting in increased appetite and food intake [37]. Increased intrauterine metabolic mediators, such as glucose, insulin, leptin, and free fatty acid, could be primarily responsible for these hypothalamic programming alterations [38]. Hypothalamic inflammation, proliferation and differentiation of neurons, as well as mitochondrial autophagy, oxidative stress and clock genes, are the primary factors involved in the process of hypothalamic programming.

### 4.1. Activation of hypothalamic inflammation and gliosis

Hypothalamic inflammation and gliosis are thought to be the foundation of hypothalamic circuit dysfunction, which can result in metabolic disorders like obesity [39]. Maternal high-fat diets (HFD) lead to lipid deposition and extensive proinflammatory gene expression in uterus. Following, inflammation factors could cross the blood–brain barrier(BBB) and act on the offspring hypothalamus to cause inflammation [40]. Hypothalamic inflammation is mainly associated with endoplasmic reticulum stress (ERS), which induces dysregulation of hypothalamic energy homeostasis. Gliosis, including astrocytes and microcytes, participates in hypothalamus dysfunction mainly by producing and releasing inflammatory factors while altering the neuronal responsiveness to metabolic signals.

Researchers observed obvious ERS in the hypothalamic neurons of offspring of high-fat diet-induced obese dams. Meanwhile, the activity of endoplasmic reticulum-associated degradation (ERAD) was reduced with a higher risk of unfolded protein response (UPR) [41]. The ERS pathway was vital for free fatty acid-induced inflammation and insulin resistance in hypothalamic neurons [42, 43]. In the hypothalamic neurons of mice born to obese dams, ERS signaling activated the pro-inflammatory c-Jun NH2-terminal kinase 1(JNK1) and nuclear factor kappa B(NF-κB) pathways and induced neural inflammation [43]. In hypothalamic neurons, the activated NF-κB pathway promoted the expression of suppressor of cytokine signaling 3 (SOCS3), which inhibited neuronal insulin and leptin signaling pathways [44]. ERS promotes the activation of autophagy during key windows of development, leading to long-term effects on hypothalamic development, which further progress to dysfunction of energy homeostasis [45].

Microglia are the tissue-resident macrophages of the central nervous system (CNS). They sense changes in microenvironment and make proper inflammatory responses [46]. In offspring of obese dams, the expression of ionized calcium binding adaptor molecule 1(IBA1), a marker of hypothalamic microglia activation, was increased [47]. Activated microglia released various pro-inflammatory factors and activated astrocytes inflammation *via* NF-κB signaling pathway [48]. More importantly, microglia sense pro-inflammatory signals associated with overeating and transmit inflammatory signals to the medial basal hypothalamus (MBH) to regulate neuronal responses to leptin and maintain energy homeostasis [49].

Astrocytes, located around blood vessels, also play a significant role in hypothalamic inflammation. They primarily take up glucose and metabolized it to lactate to supply neurons [50]. Maternal obesity during pregnancy stimulated the proliferation of astrocytes in the fetal as well as early neonatal hypothalamus, which may be driven by elevated interleukin-6 (IL-6) levels in fetal circulation [51]. Obesity during pregnancy increased fatty acid transport from the placenta to the fetus. It activated inflammatory signaling pathways in astrocytes and triggered the release of inflammatory cytokines such as IL-6, which enhanced the proliferation of astrocytes in fetal hypothalamus [38, 48, 52]. In the PVH, proliferating astrocytes alter the activity of neighboring neurons, thereby changing the energy balance and peripheral glucose metabolism [39]. Activated astrocytes also produced and released transforming growth factor-β (TGF-β). Excess TGF-β induced hypothalamic RNA stress response and mRNA metabolism-driven hypothalamic NF-κB activation which links obesity to hypothalamic inflammation [53].

Maternal over-nutrition leads to sustained hypothalamic inflammatory processes in offspring *via* interactions between neurons and non-neuronal cell populations, resulting in dysregulation of peripheral metabolism and reducing adaptive thermogenesis of brown adipose tissue [54]. Ultimately, offspring are unable to maintain the balance between caloric intake and energy expenditure, leading to overeating and further weight gain.

### 4.2. Defects in the proliferation and differentiation of hypothalamic neurons

Studies revealed that maternal obesity affects the development of offspring hypothalamic neurons by regulating neuronal proliferation. By assaying the expression of the proliferation marker Ki67 protein in rats, the offspring from obese mothers show diminished proliferation of neural progenitor cells(NPCs) [55]. In mice, the amount of cells in neurospheres generated by hypothalamic NPCs in the offspring from obese mothers is significantly lower than in controls [37]. Markers of neurogenesis and synaptic plasticity were also diminished in the hypothalamus of the offspring from obese mothers, indicating abnormal neuronal differentiation [47].

Leptin, ghrelin and insulin are crucial in neuronal axon growth. Moreover, insulin exerts direct effects on fetal neurogenesis as well [11, 23, 25, 26]. However, the hypothalamus of the offspring from obese mothers develop insulin and leptin resistance in uterus [56]. During the critical period of nervous system development, the hypothalamic neurons of the offspring from obese mothers failed to respond normally to insulin and leptin signals, thus inhibiting the axon projections of ARC neurons and significantly repressing the proliferation of NPCs [19, 24, 37]. Maternal HFD has been proven to induce the POMC neuronal malprogramming by decreasing their spatial distribution and axonal projections in ARC and PVH [57–59]. Decreases in AgRP fiber densities were also observed in the adult offspring from obese mothers due to failure to respond normally to leptin signals [58]. As a result, the plasticity of hypothalamus development is influenced and metabolic homeostasis are permanently programmed in offspring, leading to metabolic disorders in childhood and adulthood [4]. Unfortunately, although ghrelin is vital in ARC neurons projection, the evidence about alterations of ghrelin induced by maternal overnutrition influence hypothalamic development is still lacking.

Neurotrophic factors are important mediators in the differentiation and maturation of hypothalamic neurons. Studies in mice found that in the offspring of obese dams, the hypothalamic expressions of neurotrophic factors, such as BDNF and its receptor tropomyosin receptor kinase B (TrkB), were significantly reduced [47]. BDNF directly regulated synaptogenesis and neuronal plasticity in addition to its significant anorexigenic effect [60]. In addition, there was a reduction of Trk-mediated mitogen activated protein kinase (MAPK) activation in the offspring of obese dams [47]. MAPKs were localized at synaptic terminals and affected their short- and long-term plasticity by phosphorylating synaptic targets such as synaptic proteins [47]. It implies that maternal obesity affects hypothalamic neuronal plasticity in the offspring by reducing activation of MAPKs, which may be associated with a decrease in BDNF expression.

Hypothalamic neuronal differentiation is mainly regulated by the Notch pathway [61]. In the hypothalamus of the offspring born to obese dams, Notch pathway was activated, indicating by increased expression of Notch and Hes5 and decreased expression of Ngn2. The upregulation of the Notch pathway partially explained the decrease of hypothalamic NPCs proliferation [37]. Researches also pointed out that activated Notch signaling by maternal high-fat diet in the offspring’s neural stem cells altered the final differentiation and maturation processes of neurons [62]. AMPK regulates the Notch pathway by affecting the transcription of bHLH genes (such as Hes1/Hes5). In offspring exposed to maternal obesity, the levels of AMPK and pAMPK were both reduced, with the correspondingly declined regulatory function on Notch pathway [9].

### 4.3. Other findings

#### 4.3.1. Mitochondrial dysfunction

In the offspring of obese dams, hypothalamic mitochondrial oxidative phosphorylation (OXPHOS) complexes III and V were found reduced, indicating impairment of mitochondrial function. Meanwhile, hypothalamic expression levels of mitophagy markers PTEN induced putative kinase 1 (PINK1) and parkin (Prk8) were upregulated in the offspring of obese dams. It suggests that hypothalamic neurons from offspring exposed to maternal obesity exhibit mitochondrial damage and dysfunction, therefore being more prone to mitochondrial autophagy [41]. Additionally, maternal HFD programming promoted mitochondrial fusion mainly by increasing the expression of Mitofusin-2 (Mfn2) and decreasing dynamin-related protein 1 (Drp1), thereby inducing mitochondrial dysfunction [63]. The impairment of mitochondrial function may also interfere with energy metabolism and contribute to hypothalamic dysregulation of energy homeostasis.

#### 4.3.2. Oxidative stress

After birth, offspring of obese dams experience oxidative stress in the hypothalamus, resulting in defects in the function of hypothalamic appetite control neurons. Antioxidant stress reaction during the early postnatal period was elevated in offspring exposed to maternal obesity, and hypothalamic oxidative stress occurred prior to the initiation of inflammatory responses [64]. Oxidative stress occurring in the hypothalamus predisposed POMC neurons to oxidative damage and dysfunction, whereas AgRP/NPY neurons were insensitive to reactive oxygen species (ROS), hence relatively enhancing the appetite increasing effect of AgRP/NPY neurons [65, 66]. Prolonged activation of glial cells also increased the number of ROS and subsequent inflammation in hypothalamus [67].

#### 4.3.3. *Clock* genes

Maternal obesity during pregnancy may increase offspring susceptibility to obesity by affecting the daily expression pattern of the molecular clock genes and appetite genes. Circadian rhythms were regulated by CLOCK and BMAL1 transcriptional–translational feedback loops in the hypothalamic supraoptic nucleus (SCN). The feedback loop is initiated when dimer of the CLOCK and BMAL1 bond with promoter of the clock genes Period (Per) and Cryptochrome (Cry), and following the transcripts produced proteins that form dimer to repress the transcription by competing with CLOCK/BMAL1 binding [68]. The SCN neurons project to and communicate with ARC to generate circadian rhythm in feeding behaviors [69]. Maternal high-fat diet disrupted Clock inhibitory feedback pathway in offspring, leading to disruption of *Clock* gene expressions, including Per2 and Cry2. As a result, the expression pattern of appetitive peptides in the ARC was altered, and typical feeding patterns were lost, leading to obesity in adulthood [70].

## 5. Potential therapeutic approaches

Taurine deoxycholic acid (TUDCA) treatment during lactation was reported to reverse and alleviate maternal obesity-induced metabolic impairments in offspring. TUDCA, which is permeable to the blood–brain barrier (BBB), prevented the development of leptin resistance in offspring caused by maternal obesity and could reverse ERS and hyperleptinemia. More surprisingly, TUDCA also restored disrupted POMC axonal projections in offspring of obese mothers. However, POMC fiber density remained below average in adult offspring [58]. Although the detailed mechanisms of TUDCA are not fully elucidated at present, the normalization effect of hypothalamic neuronal response to metabolic stress should not be neglected [71].

Therapies targeting specific pathways have been reported but further investigations are still needed. Activation of the X-box binding protein 1(XBP1) pathway in POMC neurons reduced the expression of SOCS3 and PTP1B. It implied the potential role of XBP1 pathway in preventing hypothalamic ERS [72]. SOCS3 inhibitors or antagonists were hypothesized to have the potential to ameliorate metabolic abnormalities in offspring exposed to maternal obesity [73]. However, the effect of SOCS3 on glucose metabolism is excessively powerful, hence possible side effects must be concerned about. For instance, over-inhibition of SOCS3 leads to loss of glucose homeostasis regulation when committing physical exercise [74]. Additionally, overexpression of *Mfn2* in ARC ameliorates metabolic disturbances in diet-induced obese mice and reduces the expression of hypothalamus endoplasmic reticulum stress markers, suggesting that it could also be investigated as a therapeutic target for offspring from obese mothers [75].

## 6. Discussion

**Figure 2:**
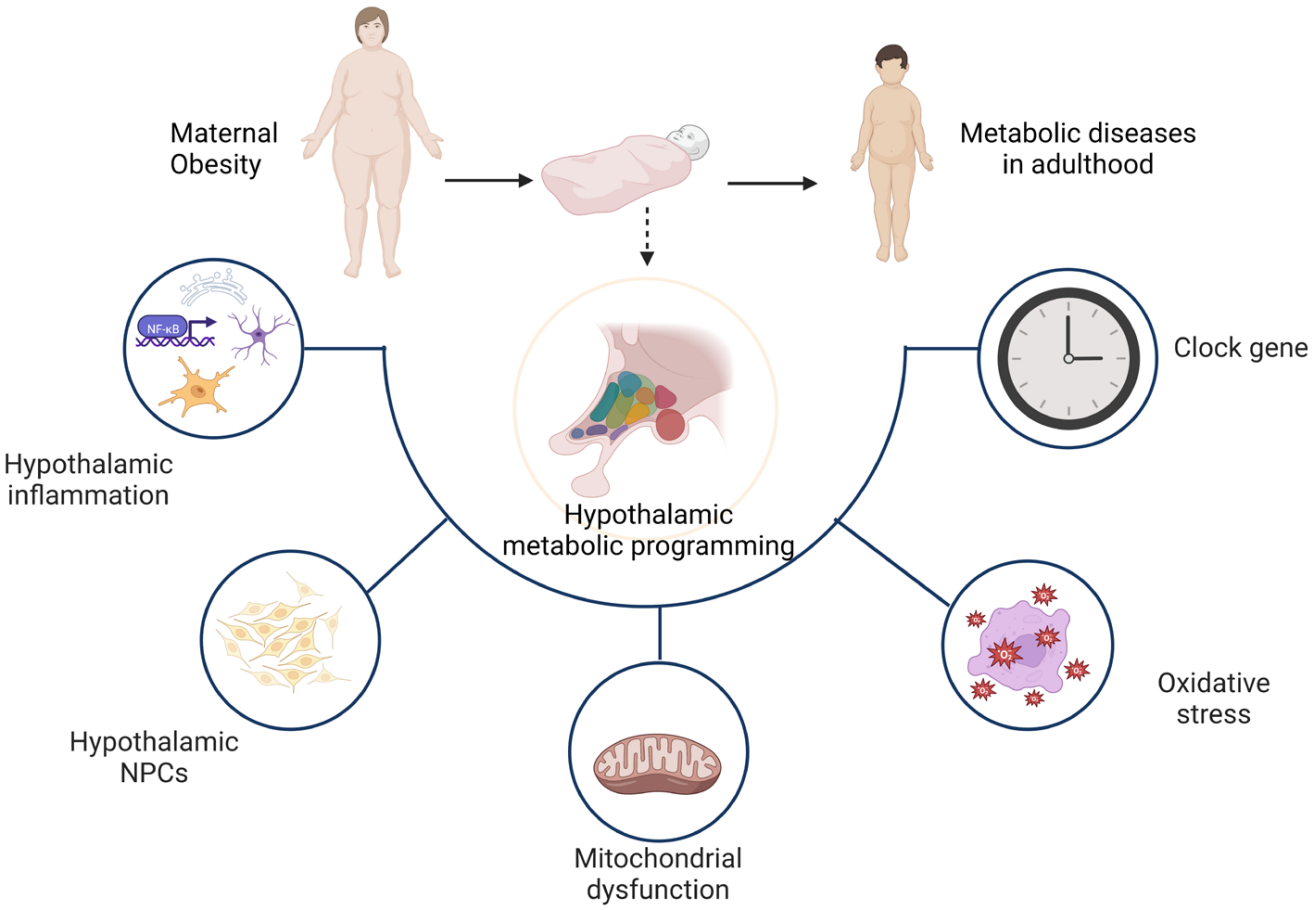
Hypothalamic programming of offspring from obese mothers mainly involves hypothalamic inflammation, neuronal proliferation and differentiation, as well as mitochondrial dysfunction, oxidative stress and clock genes. As a result, the hypothalamus shows dysfunction in the regulation of energy homeostasis and increases the likelihood of metabolic diseases later in life.

As noted previously, the hypothalamus plays a critical role in the regulation of energy homeostasis. It is a key brain region in the regulation of energy balance as it controls food intake and energy expenditure through integration of humoral, neural, and nutrient-related signals and cues. Maternal obesity programs the offspring hypothalamus mainly through hypothalamic inflammation, neuronal proliferation and differentiation, as well as mitochondrial dysfunction, oxidative stress and clock genes. Ultimately, offspring exhibit hypothalamus dysfunction in the regulation of energy homeostasis, which increases the likelihood of metabolic diseases in adulthood (Figure 2).

Studies on hypothalamus have been conducted for years, but the exact mechanisms of hypothalamic maternal programming are still not fully understood. In addition to the aforementioned mechanisms, intestinal microbiota could be another potential mechanism of offspring hypothalamic programming induced by maternal overnutrition as well. Because maternal obesity was reported to shape the microbial communities in early life of the offspring, while microbiota links obesity and hypothalamus *via* gut-brain axis [76]. Moreover, the epigenetic programming and reprogramming processes that occur during embryogenesis may be influenced by the metabolic changes caused by maternal overweight or obesity [77]. DNA methylation is both genetically and environmentally determined. In animal models, it has been shown that the prenatal and postnatal environment affects the methylation of the POMC gene, which is associated with adult weight and appetite [78]. Offspring hypermethylation in regulatory regions of POMC associated with Prenatal HFD persists until adulthood, such as promoter [79, 80]. In humans, periconceptional nutrition has been associated with offspring methylation at POMC as well [81]. Recently, a new conceptual framework of POMC neuronal heterogeneity integrating with appetite regulation, metabolic physiology and obesity was proposed [31]. It could be a new challenge for exploring hypothalamic maternal programming.

Regarding treatment and prevention, it is necessary to further investigate the mechanisms of action, the duration of maximum remission, and the long-term effects to establish their applicability in humans. The stage of hypothalamic development differs between humans and other species. In humans and primates, the development of hypothalamus is almost completed at birth, whereas in rodents, the process continues until lactation [18, 19]. Therefore, the transition of findings from animal experiments into clinical applications is the prospect to pursue.

The rate of obesity in women of childbearing age has increased steadily for decades. Maternal obesity programming has a long-term effect on the metabolism of offspring. The hypothalamus is the target brain area of metabolic programming and the regulatory center of energy metabolism. Understanding the mechanisms underlying hypothalamic regulation in the metabolism of the offspring exposed to maternal obesity is crucial for discovering novel preventive and therapeutic approaches for development origins of metabolic diseases, and is significantly important for better health outcomes in both childhood and adulthood.

## Author contributions

JZ performed the literature search and manuscript drafting. SL and XL proposed reasonable suggestions and helped revising manuscript. CZ supervised and revised the manuscript. All authors contributed to the article and approved the submitted version.

## Funding

This work was financially supported by National Natural Science Foundation of China (No. 82001605).

## Conflict of interest

The authors declare that the research was conducted in the absence of any commercial or financial relationships that could be construed as a potential conflict of interest.

## Publisher’s note

All claims expressed in this article are solely those of the authors and do not necessarily represent those of their affiliated organizations, or those of the publisher, the editors and the reviewers. Any product that may be evaluated in this article, or claim that may be made by its manufacturer, is not guaranteed or endorsed by the publisher.
